# Climate change in Switzerland: Impact on hazel, birch, and grass pollen on the basis of half a century of pollen records (1969 – 2018) 

**DOI:** 10.5414/ALX02180E

**Published:** 2020-10-12

**Authors:** Thomas Frei

**Affiliations:** Research and Consulting, Arni, Switzerland

**Keywords:** climate change, health, pollen, hay fever

## Abstract

As indicated by the Intergovernmental Panel on Climate Change (IPCC), human activities are estimated to have caused ~ 1.0 °C of global warming above pre-industrial levels. The impact of this global warming is diverse and pertains also plant biology. The start of the pollen season as well as the observed quantities of pollen have been considered indicators of the impact of climate change. Switzerland has one of the longest pollen time series now – more than half a century. It has been tested whether the impact of climate change is robust by checking with this long time series of different pollen like hazel, birch, and grass as prominent representatives of triggers of hay fever. The results indicate that based on a time series of 50 years, the pollen seasons of hazel, birch, and grass started earlier as the temperature increased. Comparing the annual amount of pollen, a relevant increase is only observed for hazel. However, it must be considered that in the observed 50 years there was a land use change from grass land to built-up land due to the increase of population, and yet certain pollen counts increased considerably.

**German version published in Allergologie, Vol. 43, No. 9/2020, pp. 357-366**

## Introduction 

International reports have documented a progressive global increase in the burden of allergic diseases across the industrialized world over the past half century. Clinical evidence reveals a general increase in both the incidence and the prevalence of respiratory diseases, including allergic rhinitis and asthma [1, 2]. Such phenomena may be related not only to air pollution and changes in lifestyle, but also to an actual increase in the amount and allergenicity of certain airborne allergenic pollen [3, 4] or prolonged pollen seasons caused by climate change [5]. 

Almost 20% of the Swiss population suffers from a pollen allergy [6]. Tree and grass pollen from wind-pollinated plants are the most important triggers [7]. The most common allergenic tree pollen in Switzerland belong to the order of the Fagales or beech family (birch, *Betula verrucosa*; alder, *Alnus glutinosa*; hazel, *Corylus avellana*; hornbeam, *Carpinus betulus*). Allergens from these pollen are highly cross-reactive. 

The global climate has changed considerably due to human activity as described by the Intergovernmental Panel on Climate Change (IPCC) [8]. On a global scale, the 5-year period 2015 – 2019 is likely to be the warmest measured, with a 1.1 °C global temperature increase since the pre-industrial period [9]. 

The impact of climate change on health has been increasingly debated and described: heat-related mortality [10, 11], vector-borne (mosquito, sandfly, and tick) and rodent-borne infections that show a trend of expanding to higher latitudes and altitudes [12, 13], or increase in hospital admissions because of flares of inflammatory bowel disease and infectious gastroenteritis during heat wave periods [14]. Finally, it has also been discussed that rising air temperatures and carbon dioxide concentrations are, in some plant species, resulting in increased pollen production and allergenicity and advancement and lengthening of the pollen season [15, 16, 17]. 

Increasing of pollen production or advancing and lengthening of the pollen season can only be reliably demonstrated by long-term measurements. The pollen trap in Basel, Switzerland, is one of the longest-existing within Europe, and the technology has been the same for the whole time period [18]. Earlier investigations with this exceptional long time series have already shown a similar trend [15, 16]. However with a time series of half a century, the earlier findings are now even more coherent. 

## Materials and Methods 

### Meteorological data 

Meteorological data were collected at the meteorological station of Basel-Binningen, 316 m above sea level. The air temperature is measured 2 m above ground according to recommendations of the World Meteorological Organization. Temperature at the climatological station Basel is measured within the automatic monitoring network of the Federal Office of Meteorology and Climatology. The station delivers a multitude of current data on the weather and climate in Switzerland every 10 minutes. The data is automatically transmitted to the central database, where various quality assurance checks are performed. 

### Pollen data 

Pollen data for Basel were collected over a period of 50 years (1969 – 2018) in a Burkard volumetric pollen and spore trap (Hirst [19]) with a sucking rate of 10 L minute^–1^. The pollen trap in Basel is located at the Kantonsspital building, 260 m above sea level. The distance between the pollen trap and the meteorological station is ~ 3 km. The start of the pollen season has been defined as the day when the sum of the daily counts reaches 2.5% of the annual pollen count, and the end of the pollen season when the sum of daily counts reaches 97.5% of the annual pollen count, whereby the pollen season encompasses 95% of the pollen recorded in the whole year [20]. There are different definitions about the start and length of the pollen season [21]. 

In the case of Corylus pollen it has to be mentioned that for the year 1975 no data were available due to technical problems with the pollen count. 

### Land use data 

Land use data have been provided by the government of canton Basel-Stadt. These data are only available since 1984 and are normally updated every 12 years. Therefore, only 4 different data points are available. 

### Statistical and graphical analyses 

Statistical analysis (t-test, Pearson correlation) and graphic presentations were carried out with the software of Microsoft Excel. 

## Results 

### Land use change 

**Figure 1.**
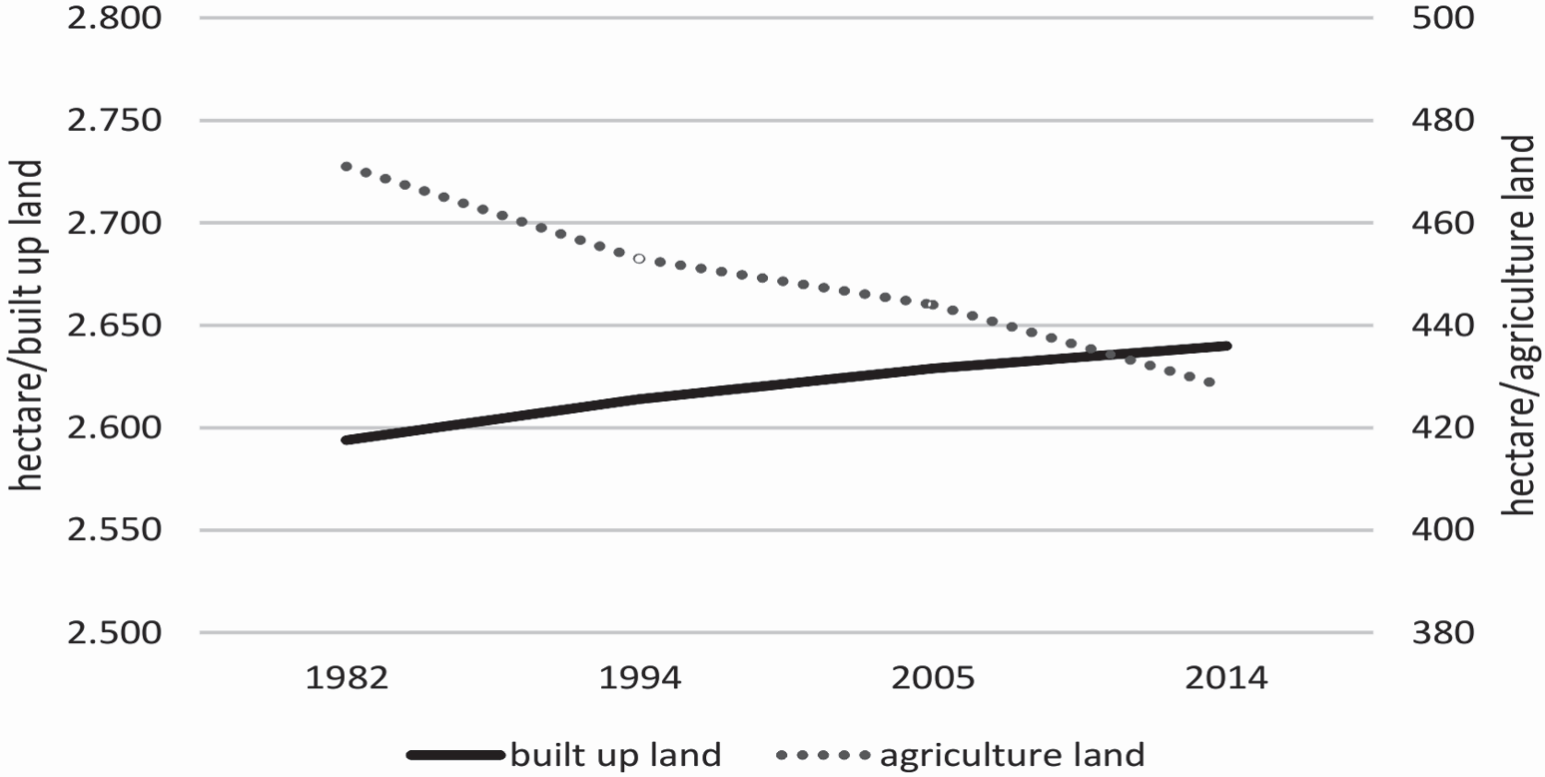
Land use change in the Kanton Basel-Stadt from 1984 to 2014.

For understanding any pattern of pollen count it was investigated whether the relation between built-up land and agricultural land had been changing over the observed time period. As shown in Figure 1, there has been a loss of agricultural land of 9% (from 471 ha to 428 ha). 

### Corylus pollen season 

**Figure 2.**
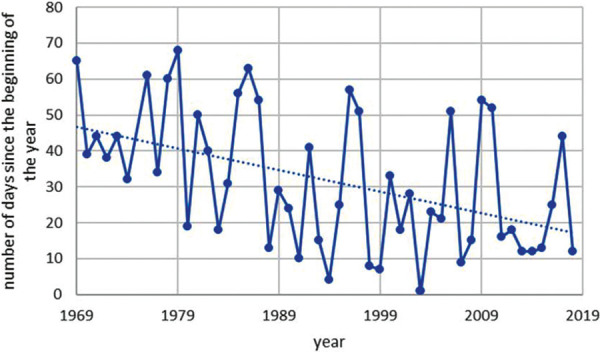
Start of Corylus pollen season in Basel.

**Figure 3.**
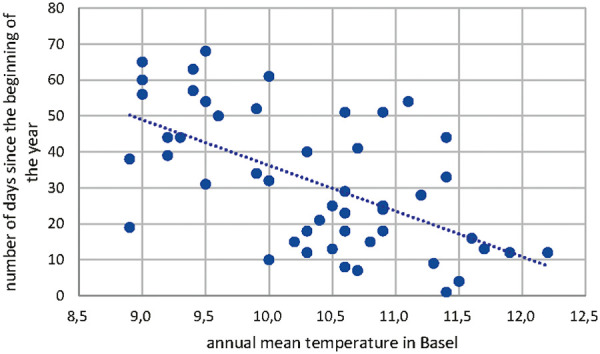
Correlation of annual mean temperature in Basel and the number of days since the beginning of Corylus pollen release.

The start of Corylus pollen season has shifted over the time period from 1969 to 2018 as shown in Figure 2. This shift has been investigated with the annual mean temperature in Basel as shown in Figure 3. The statistical analysis of this correlation between annual mean temperature and number of days since the beginning of the year results in a Pearson correlation of –0.588 with a p < 0.05 as shown in Table 1. 

**Figure 4.**
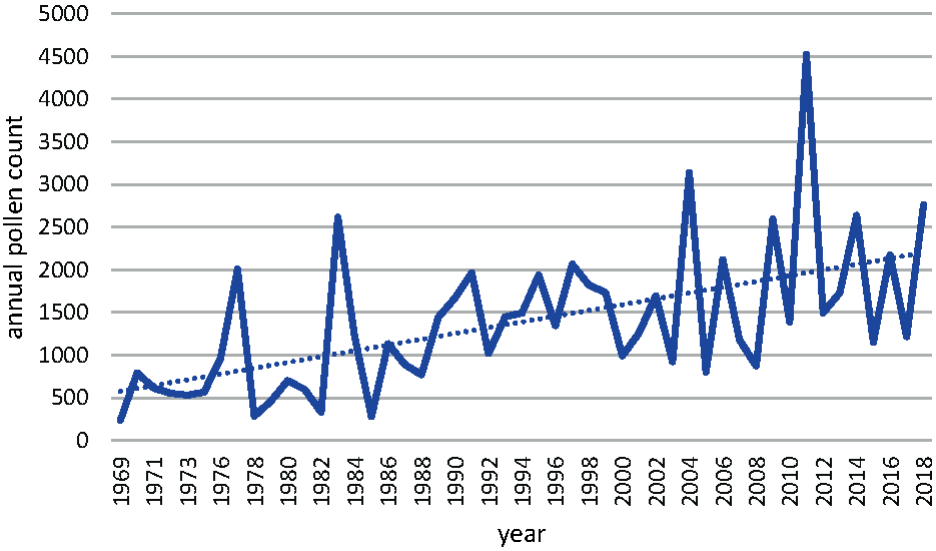
Annual Corylus pollen count in Basel.

**Figure 5.**
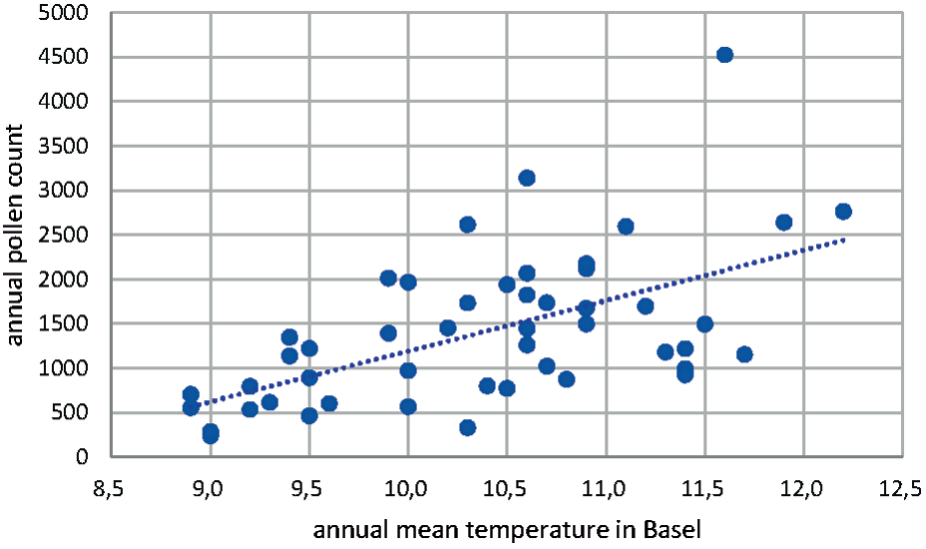
Correlation of annual mean temperature in Basel and the number of annual Corylus pollen.

The annual pollen count of Corylus in Basel also shows a shift to higher counts in the time period of 1969 – 2018 (Figure 4). The number of pollen counts increased from ~ 500 in 1969 to ~ 2,000 in 2018. The correlation of the annual mean temperature in Basel and the number of annual Corylus pollen has also been analyzed statistically and was found to be highly relevant as shown in Table 2 and Figure 5 (Pearson correlation 0.585, p < 0.05). 

### Betula pollen season 

**Figure 6:**
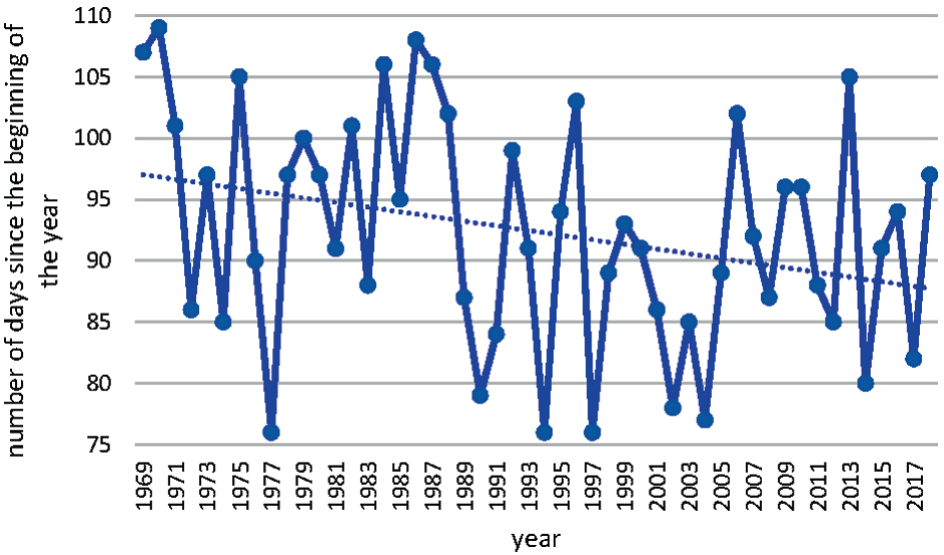
Figure 6. Start of Betula pollen season in Basel.

**Figure 7.**
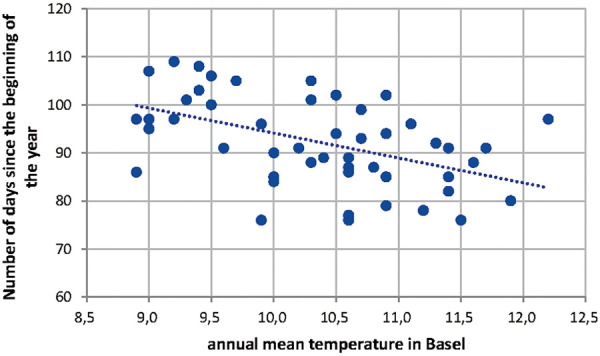
Correlation of annual mean temperature in Basel and the number of days since the beginning of Betula pollen release.

The start of Betula pollen season has shifted over the time period from 1969 to 2018 as shown in Figure 6. This shift has been investigated with the annual mean temperature in Basel as shown in Figure 7. The statistical analysis of this correlation between annual mean temperature and number of days since the beginning of the year results in a Pearson correlation of –0.485 with a p < 0.05 as shown in Table 3. 

The annual pollen count of Betula in Basel shows no shift to higher counts in the time period 1969 – 2018. 

### Poaceae pollen season 

**Figure 8.**
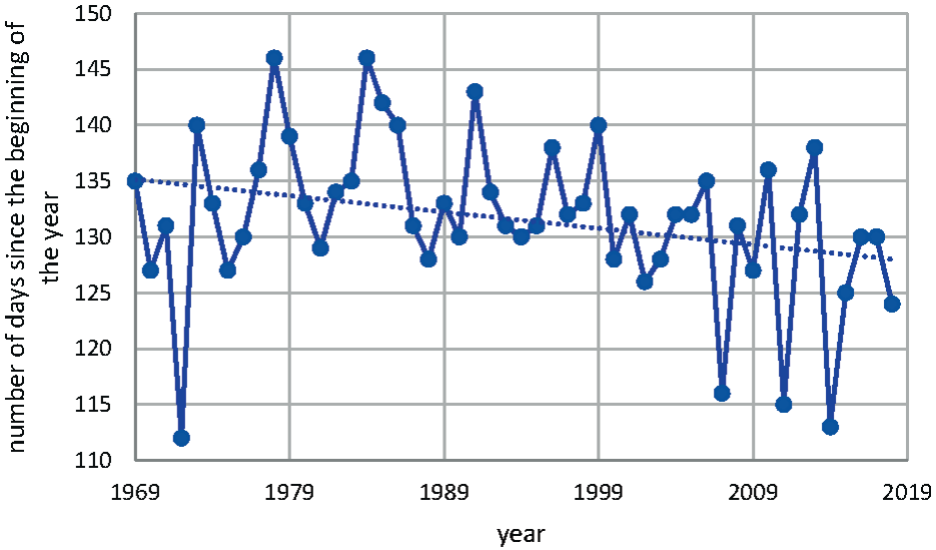
Start of Poaceae pollen season in Basel.

**Figure 9.**
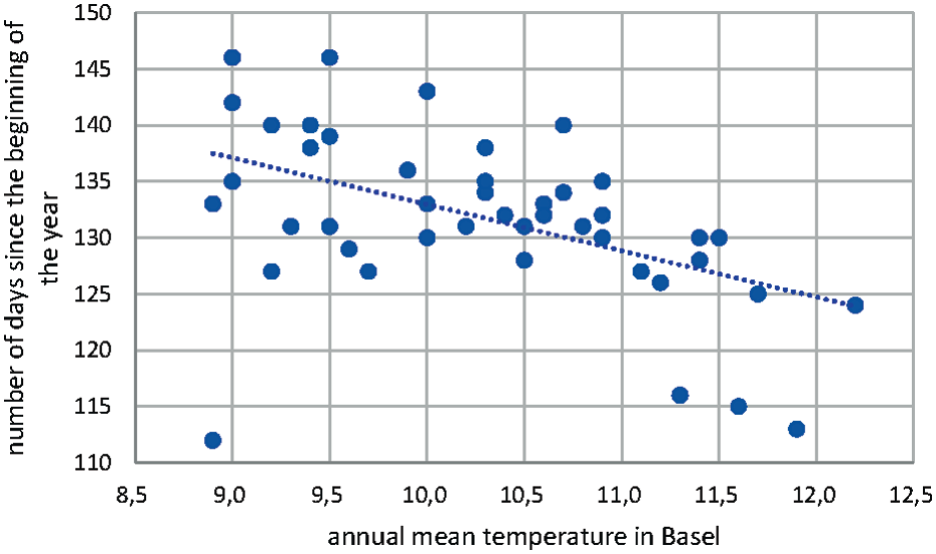
Correlation of annual mean temperature in Basel and the number of days since the beginning of Poaceae pollen release.

The start of Poaceae pollen season has shifted over the time period from 1969 to 2018 as shown in Figure 8. This shift has been investigated with the annual mean temperature in Basel as shown in Figure 9. The statistical analysis of this correlation between annual mean temperature and number of days since the beginning of the year results in a Pearson correlation of –0.493 with a p < 0.05 as shown in Table 4. 

## Conclusion 

In this study, t-test and Pearson correlation analysis have been done for the investigation of the start of the pollen season with the longest time series of pollen observation in Basel/Switzerland from 1969 to 2018. 

The main conclusions of this study are as follows:

- The start of Corylus, Betula, and Poaceae pollen season had shifted over the time period from 1969 to 2018. These shifts had been investigated with the annual mean temperature in Basel. The statistical analysis of this correlation between annual mean temperature and number of days since the beginning of the year results in a Pearson correlation of –0.588 with a p < 0.05 for Corylus, of –0.485 with a p < 0.05 for Betula, and –0.493 with a p < 0.05 for Poaceae. Between 1969 and 2018, there has been a forward shift of the beginning of the pollen season: for Corylus is in the range of 25 days, for Betula 10 days and for Poaceae 6 days.
- The annual pollen count of Corylus in Basel shows also a shift towards higher counts in the time period 1969 – 2018. The correlation of the annual mean temperature in Basel and the number of annual Corylus pollen has been analyzed statistically and found to be highly relevant (Pearson correlation 0.585, p < 0.05). The number of pollen counts increased correspondingly from 500 to 2,000.
- The increase of annual pollen count of Corylus in Basel was compared with the land use in the corresponding region. It was found, that there has been a loss of agricultural land of 9% (from 471 ha to 428 ha). That means, that the increase of pollen counts of Corylus can not be explained by a land use change but more with the increase of temperature and/or concentration of CO_2_.

## Discussion 

The longest data series from continuous volumetric pollen traps in Europe cover a period of 50 years and were obtained in Basel/Switzerland and Leiden/The Netherlands. London even started in 1961 but was interrupted between 1984 and 1987, and Stockholm started in 1973 [18]. 

Airborne pollen measurements are therefore among the longest-term datasets of biological origin, representing a valuable proxy of ongoing climate change. 

Based on data of half a century from the longest pollen data series in Switzerland (Basel 1969 – 2018), which is also one of the longest time series in Europe, the study distinctly indicates the impact of higher temperatures on earlier starting time of pollen release for all investigated plants (hazel, birch, and grass). Due to the fact of global warming [8], this means that pollen release starts earlier in hazel, birch, and grass. For the period 1969 – 2018, the shift for Corylus was in the range of 25 days between 1969 and 2018, for Betula 10 days, and for Poaceae 6 days. The influence of the higher temperature is therefore greater, the earlier in the growing season a plant begins to flower. 

Especially in the case of hazel this is of great importance for hay fever patients, since they can have symptoms already in January [22]. In the case of the released pollen amount the situation is not identical for all plants. The study showed that hazel pollen relevantly increased with increasing temperature. Therefore, global warming might have a significant impact on human health, especially in for hay fever patients. 

Extensive research over the last decade has shown that airborne pollen has increased in abundance, but pollen seasons have also shifted to an earlier timeframe and may last longer [4]. It is still not clear, though, if this is the result of increased pollen production per floral unit or per individual plant, or the consequence of land use changes, ongoing climate change, eutrophication, global warming, or a combination of these and many other factors. To date, some of the main causative factors for these changes have been considered air pollutants and higher air temperatures associated with global warming, or urbanization rates and land use changes [23]. 

## Acknowledgment 

Pollen data and meteorological data were kindly provided by the Federal Office of Meteorology and Climatology. Land use data were kindly provided by the statistical office of Kanton Basel-Stadt. 

## Funding 

The author has no funding received. 

## Conflict of interest 

The author declares no conflict of interest. 

**Table 1.** Statistical analysis of the annual mean temperature in Basel and the number of days since the beginning of Corylus pollen release.

	Variable 1	Variable 2
Mean	10.35102041	31.7755102
Variance	0.766717687	356.5110544
Observations	49	49
Pearson correlation	–0.588640907	
Hypothesized mean difference	0	
Degrees of freedom	48	
t stat	–7.726566978	
P (T<=t) one-tail	2.85788E-10	
t critical one-tail	1.677224196	
P (T<=t) two-tail	5.71575E-10	
t critical two-tail	2.010634758	

t-test: paired two sample for means.

**Table 2.** Statistical analysis of the annual mean temperature in Basel and the number of annual Corylus pollen.

	Variable 1	Variable 2
Mean	10.35102041	1391.32653
Variance	0.766717687	721248.766
Observations	49	49
Pearson correlation	0.585963474	
Hypothesized mean difference	0	
degrees of freedom	48	
t stat	–11.38947702	
P (T<=t) one-tail	1.51126E-15	
t critical one-tail	1.677224196	
P (T<=t) two-tail	3.02253E-15	
t critical two-tail	2.010634758	

t-test: paired two sample for means.

**Table 3.** Statistical analysis of the annual mean temperature in Basel and the number of days since the beginning of Betula pollen release.

	Variable 1	Variable 2
Mean	92.38	10.338
Variance	86.36285714	0.759546939
Observations	50	50
Pearson correlation	–0.485869563	
Hypothesized mean difference	0	
Degrees of freedom	49	
t Stat	59.52181264	
P (T<=t) one-tail	1.15075E-47	
t critical one-tail	1.676550893	
P (T<=t) two-tail	2.3015E-47	
t critical two-tail	2.009575237	

t-est: paired two sample for means.

**Table 4.** Statistical analysis of the annual mean temperature in Basel and the number of days since the beginning of Poaceae pollen release.

	Variable 1	Variable 2
Mean	131.58	10.338
Variance	53.06489796	0.759546939
Observations	50	50
Pearson correlation	–0.493121532	
Hypothesized mean difference	0	
Degrees of freedom	49	
t stat	110.5992921	
P (T<=t) one-tail	9.50237E-61	
t Critical one-tail	1.676550893	
P (T<=t) two-tail	1.90047E-60	
t critical two-tail	2.009575237	

t-test: paired two sample for means.
